# Study of Non-invasive Laboratory and Imaging Predictors of Large Oesophageal Varices in Patients With Liver Cirrhosis at an Industrial Hospital in Eastern India

**DOI:** 10.7759/cureus.104681

**Published:** 2026-03-04

**Authors:** Sangita D Kamath, Raghavpudi S Chandra, Manish Kumar

**Affiliations:** 1 Internal Medicine, Tata Main Hospital, Jamshedpur, IND

**Keywords:** cirrhosis, non-invasive predictors, oesophageal, portal hypertension, varices

## Abstract

Introduction

Oesophageal variceal bleeding, particularly from large varices, remains a major driver of morbidity and mortality in liver cirrhosis (LC), highlighting the importance of early detection of clinically significant varices. Because universal endoscopic screening may be difficult to implement in resource-limited settings, we evaluated readily available non-invasive laboratory and ultrasonographic predictors of large oesophageal varices (EV) in patients with cirrhosis.

Aim

This study aims to assess the utility of non-invasive laboratory and ultrasonographic parameters in predicting the severity of large EV in patients with LC treated at an industrial hospital in Eastern India.

Methods

We conducted a prospective observational study of newly diagnosed patients aged 15 years and older with LC at Tata Main Hospital, Jamshedpur, Jharkhand, India. We enrolled 156 patients evaluated between March 2023 and February 2025, and all participants underwent clinical assessment, laboratory testing, abdominal ultrasonography with Doppler, and upper gastrointestinal endoscopy (UGIE) for EV detection and grading. We evaluated associations between clinical, laboratory, and imaging variables and EV severity and used receiver operating characteristic (ROC) analysis to identify cut-off values for predicting large EV and performed internal validation of these cut-offs using bootstrap resampling.

Results

The study included 156 patients with LC with a mean age of 59.2 ± 11.0 years; most patients were 40 to 59 years old (69.2%, 83/156). The cohort included 121 men (77.6%) and 35 women (22.4%), and alcohol use was the most common aetiology (35.9%, 56/156). UGIE showed EV in 144/156 patients (92.3%) and no EV in 12/156 patients (7.7%); grade III EV was most common (49.4%, 77/156), and grade IV EV occurred in 5.1% (8/156). In multivariate ordinal logistic regression, predictors of variceal severity included Child-Turcotte-Pugh score (p < 0.0001), Model for End-Stage Liver Disease score (p = 0.003), platelet count (p < 0.0001), international normalized ratio (p = 0.002), aspartate aminotransferase-to-platelet ratio index (APRI; p < 0.0001), platelet count/spleen diameter ratio (PC/SD; p < 0.0001), portal vein (PV) diameter (p < 0.0001), and anaemia (p = 0.029). For predicting large EV, ROC analysis showed excellent discrimination for APRI (area under the curve (AUC) 0.93; cut-off > 2.11; sensitivity 89.4%; specificity 78.9%; positive predictive value (PPV) 83.5%; negative predictive value (NPV) 86.2%; diagnostic accuracy (DA) 84.6%) and PC/SD (AUC 0.93; cut-off ≤ 855; sensitivity 81.7%; specificity 91.5%; PPV 92%; NPV 80.2%; DA 85.9%). Additional predictors included aspartate aminotransferase/alanine aminotransferase ratio (AUC 0.92; cut-off > 1.2) and platelet count (AUC 0.91; cut-off ≤ 41,000).

Conclusion

EV were common in this high‑risk, tertiary cohort of patients with LC, and clinically significant varices were frequently identified on endoscopy. Among the non-invasive measures evaluated, the APRI and the platelet count-to-spleen diameter ratio showed the strongest overall performance for identifying patients at risk of large EV. These readily available indices, supported by ultrasonographic measures such as PV diameter, may help prioritize endoscopy for patients at high risk, particularly in settings with limited endoscopic capacity, pending external validation and threshold updating.

## Introduction

Liver cirrhosis (LC) represents an advanced stage in the spectrum of chronic liver disease (CLD) and is characterized by disruption of normal hepatic parenchymal architecture, deposition of extracellular matrix, formation of regenerative nodules, vascular neogenesis, and development of portal hypertension. Cirrhosis is a major global health concern and is the 15th leading cause of disability-associated life years worldwide [1]. The estimated incidence of cirrhosis is 23.6/100,000 in Southeast Asia [2]. Data from the Global Burden of Disease Study indicate an age-adjusted death rate for LC of 7.96 per 100,000 population [3].

One of the most clinically significant consequences of portal hypertension is the development of oesophageal varices (EV). EV occur in approximately 60% of patients with decompensated liver disease and 40% of patients with compensated LC [4,5]. Variceal bleeding carries a high risk of mortality, underscoring the importance of early detection and prophylactic management. In a study by Wang et al., variceal bleeding accounted for 28.9% of deaths among patients with cirrhosis [6]. The annual incidence of new varices is approximately 7% to 8% [7,8]. In addition, small varices progress to large varices at a rate of 7% to 10% per year, and the annual risk of bleeding is 5% to 15% [8]. Mortality following bleeding from EV is approximately 20% at six weeks [7,9]. The risk of bleeding is highest in patients with large EV, consistent with higher variceal wall tension [7].

Upper gastrointestinal endoscopy (UGIE) remains the gold standard for detecting EV in patients with LC. The Baveno VI Meeting Consensus recommends that all patients with LC undergo screening endoscopy for EV at diagnosis and at regular intervals thereafter [10]. Although UGIE is generally safe when performed by experienced clinicians, it carries a small risk of complications, including perforation, infection, and bleeding. In addition, some older adults have difficulty tolerating or adhering to the procedure. Endoscopy also increases healthcare costs and may not be readily available in all settings. These barriers are particularly relevant in resource-limited settings such as India, where restricting UGIE to patients at the highest risk for large EV may be more cost-effective. Accordingly, there is a need for non-invasive markers that can predict the presence of large EV in patients with LC.

Several studies have evaluated non-invasive predictors of large EV. Reported markers include low platelet count [8,11-14], advanced Child-Pugh score [5,8,12], Model for End-Stage Liver Disease (MELD) score [15], splenomegaly [14,16], aspartate aminotransferase-to-alanine aminotransferase (AST/ALT) ratio (AAR) [17], AST-to-platelet ratio index (APRI) [17,18], serum albumin [19], and imaging measures such as increased portal vein (PV) diameter [11,20,21] and splenic bipolar diameter [14,22]. Prior work has also supported the ratio of platelet count (×10^3^/µL) to maximum splenic bipolar diameter (PC/SD ratio) as a predictor of large EV [8,11,23]. Because these indices rely on routinely obtained laboratory and imaging parameters used in the initial evaluation of LC, they are widely accessible and practical for clinical use. However, the performance of individual markers varies across populations, likely due to differences in cirrhosis aetiology and disease stage. Industrial hospitals, which serve heterogeneous patient populations with diverse aetiologies of liver disease, represent an appropriate setting for evaluating these predictors.

With this background, this study aimed to assess the utility of non-invasive laboratory and imaging parameters in predicting the presence and severity of EV in patients with cirrhosis treated at an industrial hospital in Eastern India. By identifying simple, widely available indices and imaging measures, this study seeks to support a pragmatic and cost-effective screening approach that optimizes the use of endoscopic resources while enabling timely detection of clinically significant varices.

## Materials and methods

This prospective observational study was conducted in the outpatient and inpatient departments of Medicine and Gastroenterology at Tata Main Hospital (TMH), Jamshedpur, Jharkhand, India, between March 2023 and February 2025. TMH is a 985-bed multidisciplinary industrial hospital. The Institutional Ethics Committee approved the study (approval No. TMH/IEC/FEB/074/2023), and all participants provided written informed consent.

We calculated the sample size using a single-proportion, precision-based approach: $n=[Z1-\alpha/2]^{2}x[P(1-P)]/d^{2}$, where $Z1-\alpha/2$ = 1.96 for a two-sided 95% confidence level, P is the anticipated EV prevalence in the target cohort, and d is the desired absolute precision. Using P = 0.611 (based on prior local data in this tertiary referral setting) and d = 0.08, the initial estimate was n ≈ 142.

To allow for ~10% attrition/non-evaluable cases, we inflated the target sample size to ≈158 as follows: $n0=n/1-0.10\approx 142/0.90\approx 158$, and the final enrolled sample was 156.

Because this was a tertiary industrial hospital with endoscopy availability and a high proportion of symptomatic referrals, the enrolled cohort likely included a greater proportion of patients with decompensated cirrhosis than would be expected in community-based screening populations.

The study included newly diagnosed patients aged 15 years or older with a diagnosis of LC, irrespective of aetiology, who provided informed consent to participate. We enrolled eligible patients consecutively during the study period.

We excluded patients with active variceal bleeding or a prior history of bleeding, including those who had previously undergone sclerotherapy or band ligation of EV. We also excluded patients with PV thrombosis, hepatocellular carcinoma, and current or past treatment with beta-adrenergic receptor blockers.

All participants underwent comprehensive clinical evaluation, laboratory testing, imaging studies, and UGIE at our centre. The clinical history included demographic characteristics (age and sex), presenting symptoms, and a focused assessment for haematemesis, melena, bleeding per rectum, bleeding diathesis, and jaundice. We also assessed lifestyle factors and relevant medical history, including alcohol intake and duration, prior blood transfusions, use of hepatotoxic drugs, exposure to sexually transmitted diseases, and intravenous drug use. Clinicians performed a physical examination and documented anaemia and complications of CLD, including splenomegaly, ascites, icterus, encephalopathy, and other signs of liver failure. We defined and graded hepatic encephalopathy (HE) using the West Haven classification system [24].

Laboratory testing included complete blood count; renal function tests (blood urea and serum creatinine); and liver function tests (serum bilirubin, ALT, AST, alkaline phosphatase, total serum proteins, serum albumin, globulin, prothrombin time, and international normalized ratio (INR)). Using these laboratory values, we calculated non-invasive indices for each patient, including the AST/ALT ratio, APRI, PC/SD ratio, and MELD score.

Calculation of non-invasive markers

APRI was calculated as APRI = ((AST/AST (upper limit of normal) IU/L) × 100)/platelet count, where the AST upper limit of normal was 40 IU/L (local laboratory reference). Platelet count was expressed as n ×10^3^/µL for APRI calculations.

The PC/SD ratio was calculated as platelet count (per µL)/spleen bipolar diameter (mm), consistent with prior literature; spleen diameter was recorded in millimetres on ultrasonography. The MELD score was calculated using the United Network for Organ Sharing (UNOS) MELD calculator [25].

Imaging included abdominal ultrasonography and Doppler evaluation of the hepatic veins and PVs. We recorded liver echotexture and size; splenic enlargement (bipolar diameter in millimetres); PV diameter; presence of ascites; direction of blood flow; thrombosis; and the presence of collateral vessels.

PV Doppler studies were performed using a subcostal/intercostal approach with B‑mode, colour Doppler, and spectral Doppler, with the patient fasting for ≥4 hours and measurements obtained during quiet respiration. Spectral Doppler settings included an insonation Doppler angle ≤60°, a low wall filter, and a low-to-moderate pulse repetition frequency to capture low‑velocity hepatopetal flow. All measurements were obtained by the same radiologist.

UGIE was performed for all patients in the same endoscopy unit using an Olympus CV‑190 video endoscope (Olympus Corporation, Tokyo, Japan). We recorded the presence and grade of EV, portal hypertensive gastropathy, and duodenopathy. Varices were graded using Paquet classification under moderate insufflation to grade EV and categorized as large (Grade III-IV) or small (Grade I-II) [26]. UGIE was performed within 48 hours of admission for all patients by the same gastroenterologist, who was blinded to clinical data, laboratory results, and imaging parameters.

To evaluate the aetiology of cirrhosis, we obtained viral markers, including hepatitis B surface antigen and anti-hepatitis C virus antibody assay. When indicated, we performed testing for autoimmune and metabolic causes of liver disease, including serum ceruloplasmin, slit-lamp examination, antinuclear antibody, anti-smooth muscle antibody, anti-liver kidney muscle antibody, and anti-mitochondrial antibody assay. We diagnosed chronic drug‑induced liver injury (DILI) or herb‑induced liver injury (HILI), based on a documented history of exposure to the implicated agent together with exclusion of viral, autoimmune, metabolic, and other alternative causes. We diagnosed LC based on clinical and ultrasonographic findings, and we classified the severity of liver dysfunction using the modified Child-Turcotte-Pugh (CTP) score [27].

Statistical analysis

We analyzed data using IBM SPSS Statistics for Windows, Version 22 (Released 2013; IBM Corp., Armonk, New York). Categorical variables are presented as frequencies and proportions. Continuous variables are presented as mean ± SD when approximately normally distributed and as median (IQR) when skewed. We compared variables across variceal-grade groups (no EV, small EV, large EV) using analysis of variance for approximately normal variables and the Kruskal-Wallis test for skewed variables; categorical variables were compared using Pearson’s chi-square test. For diagnostic classification, we evaluated performance for large EV (grade III-IV) versus non-large EV (grade I-II) or no EV using receiver operating characteristic (ROC) curve analysis. We reported area under the curve (AUC) values with 95% confidence intervals (CIs), identified optimal cut-offs using Youden’s J statistic, and calculated sensitivity and specificity at selected cut-offs. Because across-grade group comparisons and binary ROC discrimination assess different hypotheses, their p-values and effect patterns are not expected to be concordant, particularly for skewed biomarkers. We examined distributions for extreme values and confirmed that selected ROC cut-offs were not driven by isolated outliers. To quantify uncertainty around sensitivity and specificity at each cut-off, we generated bootstrap 95% CIs using 2,000 stratified replicates (stratified by large vs. non-large EV). A two-sided p-value < 0.05 was considered statistically significant. We interpreted AUC values as follows: 0.9-1, excellent; 0.8-0.9, good; 0.7-0.8, fair; 0.6-0.7, poor; and 0.5-0.6, fail [28].

Variables with statistically significant associations in univariable analyses were entered into ordinal logistic regression models to identify independent predictors of variceal severity, and we report odds ratios (ORs) with 95% CIs. The outcome was coded such that higher categories represented greater variceal severity; therefore, ORs >1 indicate increased odds of a higher variceal grade.

We evaluated the proportional odds (parallel lines) assumption using a Brant-style screening approach (threshold-specific binary logits with a Wald test of slope equality) and found no evidence of violation (global χ² = 11.90, df = 6, p = 0.064; all variable-specific screening tests p > 0.20). A formal Brant test in R/Stata was prespecified for confirmation. We assessed potential collinearity, particularly among platelet-based indices (platelet count, APRI, PC/SD), using Spearman correlations and variance inflation factors. Based on these assessments, APRI was prespecified for the primary model, and platelet count and PC/SD were evaluated in sensitivity specifications, which yielded consistent inferences.

## Results

The study included 156 cases of LC observed between March 2023 and February 2025 in the Departments of Gastroenterology and Medicine at Tata Main Hospital. The mean age was 59.2 ± 11.0 years, and most patients (69.2%, n=83) were 40 to 59 years of age (range, 19 to 80 years). Age showed a significant inverse gradient with variceal severity; median age fell from 73.5 (no EV) to 53.5 years (large EV; p = 2.54×10⁻⁶). The cohort included 121 men (77.6%) and 35 women (22.4%), yielding a male-to-female ratio of 6.5:1. Alcohol use was the most common aetiology, affecting 56 patients (35.9%). Table 1 summarizes the demographic characteristics and distribution of other aetiological factors.

**Table 1:** Aetiology of liver cirrhosis in the study population (n=156)

Aetiology	Number	Percentage (%)
Alcohol	56	35.9
Hepatitis B virus (HBV)	26	16.7
Hepatitis C virus (HCV)	23	14.7
Cryptogenic	31	19.9
Drugs (including herbal)	20	12.8

Clinical findings are summarized in Table 2. Pallor was present in 102 (65.4%) patients, jaundice in 78 (50%), pedal oedema in 125 (80.1%), and spider naevi in 34 (21.8%). Ascites was absent in 72 (46.15%) patients; mild ascites was present in 42 (26.9%), moderate ascites in 31 (19.87%), and gross ascites in 11 (7.1%). HE was present in 27 (17.3%) patients; among those with HE, 55.5% had grade 1 HE, and grade IV HE occurred in 14.8% of cases. Splenomegaly was observed in 78 (50%) patients. Based on the modified CTP classification, most patients (n=81, 51.9%) were in CTP class C, indicating decompensated cirrhosis; 48 (30.8%) were in CTP class B, and 27 (17.3%) were in CTP class A.

**Table 2:** Clinical features of the study population (n=156)

Clinical feature	Number of patients	Percentage (%)
Pallor	102	65.4%
Jaundice	78	50.0%
Pedal oedema	125	80.1%
Spider naevi	34	21.8%
Ascites: none	72	46.2%
Ascites: mild	42	26.9%
Ascites: moderate	31	19.9%
Ascites: gross	11	7.1%
Hepatic encephalopathy (grade I–IV)	27	17.3%
Splenomegaly	78	50.0%

Mean baseline laboratory parameters are shown in Table 3. Anaemia occurred in 116 patients (74.4%), thrombocytopenia in 81 (51.9%), and neutropenia in 17 (10.9%). The mean serum sodium level was 130.12 ± 2.9 mmol/L, while that of potassium was 4.5 ± 0.9 mmol/L. The mean blood urea level was 89.38 ± 15.4, and that of the serum creatinine level was 1.37 ± 0.5 mg/dL. Mean serum albumin was 2.43 ± 0.95 g/dL, and mean total bilirubin was 2.82 ± 1.2 mg/dL.

**Table 3:** Baseline laboratory parameters of the study population (n=156) Abbreviation: SD, standard deviation

Laboratory parameter	Mean ± SD
Haemoglobin (g/dL)	9.2 ± 2.4
White blood cell count (/cu mm)	8.2 ± 4.4
Platelet count (×10^3^/µL)	96.3 ± 25.5
Total bilirubin (mg/dL)	2.8 ± 1.2
Alanine aminotransferase (IU/L)	44.2 ± 8.9
Aspartate aminotransferase (IU/L)	52.5 ± 9.2
Serum albumin (g/dL)	2.4 ± 0.9
Blood urea (mg/dL)	89.4 ± 15.4
Serum creatinine (mg/dL)	1.4 ± 0.5
Serum sodium (mmol/L)	130.1 ± 2.9
Serum potassium (mmol/L)	4.5 ± 0.9

Table 4 summarizes baseline UGIE findings. Twelve patients (7.7%) had no evidence of EV, whereas 144 had EV ranging from grade 1 to grade IV. Grade III varices were the most common finding (77, 49.4%), and eight (5.1%) patients had grade IV varices. Portal hypertensive gastropathy and gastric antral vascular ectasia (GAVE) were present in 58.3% and 8.3% of cases, respectively. The distribution of cirrhosis severity in this cohort (with a predominance of advanced disease) likely contributed to the high prevalence of EV. This prevalence should be interpreted in the context of a referral-based hospital population rather than a population-level screening sample.

**Table 4:** Upper gastrointestinal endoscopy findings in patients with liver cirrhosis (n=156) Abbreviations: PHG, portal hypertensive gastropathy; GAVE, gastric antral vascular ectasia.

Category	Finding	No.(%) of patients
Oesophageal varices	Grade I	24 (15.4%)
	Grade II	35 (22.4%)
	Grade III	77 (49.4%)
	Grade IV	8 (5.1%)
Other findings	Gastric erythema/erosions	23 (14.7%)
	PHG	91 (58.3%)
	GAVE	13 (8.3%)
	Portal hypertensive duodenopathy	17 (10.9%)
Oesophageal varices absent	Normal study	12 (7.7%)

The relationship between clinical, laboratory, and ultrasonographic variables and EV severity is summarized in Table 5. Median age decreased with variceal severity, from 73.5 years (IQR, 69.0-78.0) in patients without varices to 53.5 years (IQR, 47.0-63.5) in those with large varices. The proportion of men also increased across severity groups, from seven men in the no-varices group to 70 men in the large-varices group; however, the sex distribution did not differ significantly across variceal-grade groups (p = 0.143). Liver function worsened with increasing variceal size, with median total bilirubin increasing from 0.78 mg/dL (IQR, 0.705-1.088) in patients without varices to 2.95 mg/dL (IQR, 1.93-3.68) in those with large varices (p = 3.96×10⁻¹¹) and serum albumin decreasing from 3.07 g/dL (IQR, 2.88-3.53) to 2.51 g/dL (IQR, 2.13-2.78) (p = 3.20×10⁻⁵). ALT and AST also differed significantly across groups (p = 0.022 and p < 0.001, respectively). Haematologic parameters worsened with increasing EV severity: median haemoglobin decreased from 10.0 g/dL (IQR, 9.33-10.30) in patients without varices to 7.85 g/dL (IQR, 6.90-8.88) in patients with large EV (p = 3.97×10⁻⁵), and median platelet count decreased from 112.5 ×10^3^/µL (IQR, 106.8-182.3) to 87.0 ×10^3^/µL (IQR, 65.1-102.8) (p = 0.00180). INR increased from a median of 1.25 (IQR, 1.135-1.30) in the no-varices group to 1.80 (IQR, 1.51-2.13) in the large-varices group (p = 1.26×10⁻¹⁰). Serum sodium decreased from 133.54 ± 3.21 mmol/L to 125.41 ± 2.41 mmol/L with increasing disease severity (p < 0.001). Both CTP and MELD scores increased significantly with higher variceal grades: the median CTP score increased from 7.0 (IQR, 6.0-8.0) to 11.0 (IQR, 10.0-12.75), and the median MELD score increased from 10.5 (IQR, 9.75-12.0) to 19.0 (IQR, 15.0-26.75) (p = 1.63×10⁻¹⁰). The median AAR and APRI also increased with variceal severity. Markers associated with portal hypertension changed significantly: the median PC/SD ratio decreased from 914.0 (IQR, 864.4-1352.2) in patients without varices to 603.9 (IQR, 466.9-693.4) in those with large varices (p = 2.59×10⁻⁵), while the median PV diameter increased from 12.65 mm (IQR, 12.23-12.90) to 14.6 mm (IQR, 14.20-15.00) (p = 6.84×10⁻¹⁵).

**Table 5:** Demographic, laboratory, and ultrasonographic parameters by oesophageal variceal grade (Paquet classification) Values are median (IQR) unless otherwise indicated (*mean ± SD). P-values were obtained using the Kruskal–Wallis test for median (IQR) variables and one-way ANOVA for *mean ± SD variables. Sex was compared using the χ^2^ test or Fisher's exact test, as appropriate. When the Kruskal–Wallis omnibus p-value was <0.05, Dunn pairwise tests with Holm correction were applied. Abbreviations: ANOVA, analysis of variance; AAR, AST/ALT ratio; ALT, alanine aminotransferase; APRI, AST-to-platelet ratio index; AST, aspartate aminotransferase; CTP, Child–Turcotte–Pugh; EV, oesophageal varices; INR, international normalized ratio; IQR, interquartile range; MELD, Model for End-Stage Liver Disease; PC/SD, platelet count-to-spleen diameter ratio; WBC, white blood cell count.

Variable	No EV (n = 12)	Small EV (Paquet I–II; n = 59)	Large EV (Paquet III–IV; n = 85)	P-value
Sex, male, n (%)	7 (58.3)	44 (74.6)	70 (82.4)	0.143
Sex, female, n (%)	5 (41.7)	15 (25.4)	15 (17.6)	0.143
Age, years	73.5 (69.0–78.0)	62.5 (55.3–68.0)	53.5 (47.0–63.5)	2.54 × 10^-6^
Haemoglobin, g/dL	10.0 (9.33–10.30)	8.70 (7.83–9.60)	7.85 (6.90–8.88)	3.97 × 10^-5^
WBC, ×10^3^/µL	7.55 (6.14–8.54)	6.11 (4.48–8.09)	5.30 (3.98–7.64)	0.113
Platelet count, ×10^3^/µL	112.5 (106.8–182.3)	97.1 (76.0–108.0)	87.0 (65.1–102.8)	0.00180
Albumin, g/dL	3.07 (2.88–3.53)	2.75 (2.49–2.93)	2.51 (2.13–2.78)	3.20 × 10^-5^
Total bilirubin, mg/dL	0.78 (0.705–1.088)	1.48 (1.01–1.988)	2.95 (1.93–3.68)	3.96 × 10^-11^
INR	1.25 (1.135–1.30)	1.38 (1.29–1.61)	1.80 (1.51–2.13)	1.26 × 10^-10^
ALT, IU/L*	41.2 ± 10.5	47.2 ± 9.8	44.1 ± 6.5	0.022
AST, IU/L*	47.8 ± 7.6	58.5 ± 9.8	51.2 ± 10.2	<0.001
AAR	1.64 (1.60–1.96)	1.86 (1.51–2.32)	2.26 (1.88–3.05)	0.00075
APRI	0.70 (0.55–0.80)	1.10 (0.80–1.55)	1.40 (1.02–2.28)	8.09 × 10^-5^
PC/SD (platelets ÷ spleen diameter)	914.0 (864.4–1352.2)	689.5 (519.7–760.0)	603.9 (466.9–693.4)	2.59 × 10^-5^
Portal vein diameter, mm	12.65 (12.23–12.90)	13.80 (13.50–14.10)	14.60 (14.20–15.00)	6.84 × 10^-15^
CTP score	7.0 (6.0–8.0)	9.0 (8.0–9.0)	11.0 (10.0–12.75)	5.73 × 10^-15^
MELD score	10.5 (9.75–12.0)	13.0 (11.0–15.75)	19.0 (15.0–26.75)	1.63 × 10^-10^
Sodium, mmol/L*	133.54 ± 3.21	131.41 ± 3.11	125.41 ± 2.41	<0.001

Table 6 presents the multivariate ordinal logistic regression analysis used to identify independent predictors of variceal severity. The CTP score (p < 0.0001), MELD score (p = 0.003), platelet count (p < 0.0001), INR (p = 0.002), APRI score (p < 0.0001), PC/SD ratio (p < 0.0001), PV diameter in mm (p < 0.0001), and anaemia (p = 0.029) were statistically significant independent predictors. Total bilirubin (p = 0.069), albumin (p = 0.075), and age (p = 0.054) showed borderline significance.

**Table 6:** Multivariate binary logistic regression analysis for large oesophageal varices (Paquet grade III–IV) *From multivariate ordinal logistic regression. aORs are from a multivariable binary logistic regression model including all predictors shown; aOR > 1 indicates higher odds of a higher variceal grade, and aOR < 1 indicates lower odds. Abbreviations: aOR, adjusted odds ratio; CI, confidence interval; CTP, Child–Turcotte–Pugh; MELD, Model for End‑Stage Liver Disease; INR, international normalized ratio; APRI, AST-to-platelet ratio index; PC/SD, platelet count-to-spleen diameter ratio; PV, portal vein; Hb, haemoglobin.

Predictor	aOR	Asymptotic 95% CI (lower–upper)	P-value*
Age	1.057	0.998–1.120	0.054
CTP score	1.20	1.11–1.30	<0.0001
MELD score	1.52	1.30–1.77	0.003
Platelet count	0.904	0.856–0.954	<0.0001
Serum albumin	0.679	0.438–1.037	0.075
Total bilirubin	1.339	0.984–1.854	0.069
INR	1.55	1.33–1.81	0.002
APRI	1.08	1.03–1.13	<0.0001
PC/SD ratio	0.928	0.888–0.969	<0.0001
Portal vein (PV) diameter	1.26	1.15–1.39	<0.0001
Anemia (Hb < 10 g/dL)	2.71	1.12–6.76	0.029

**Figure 1:**
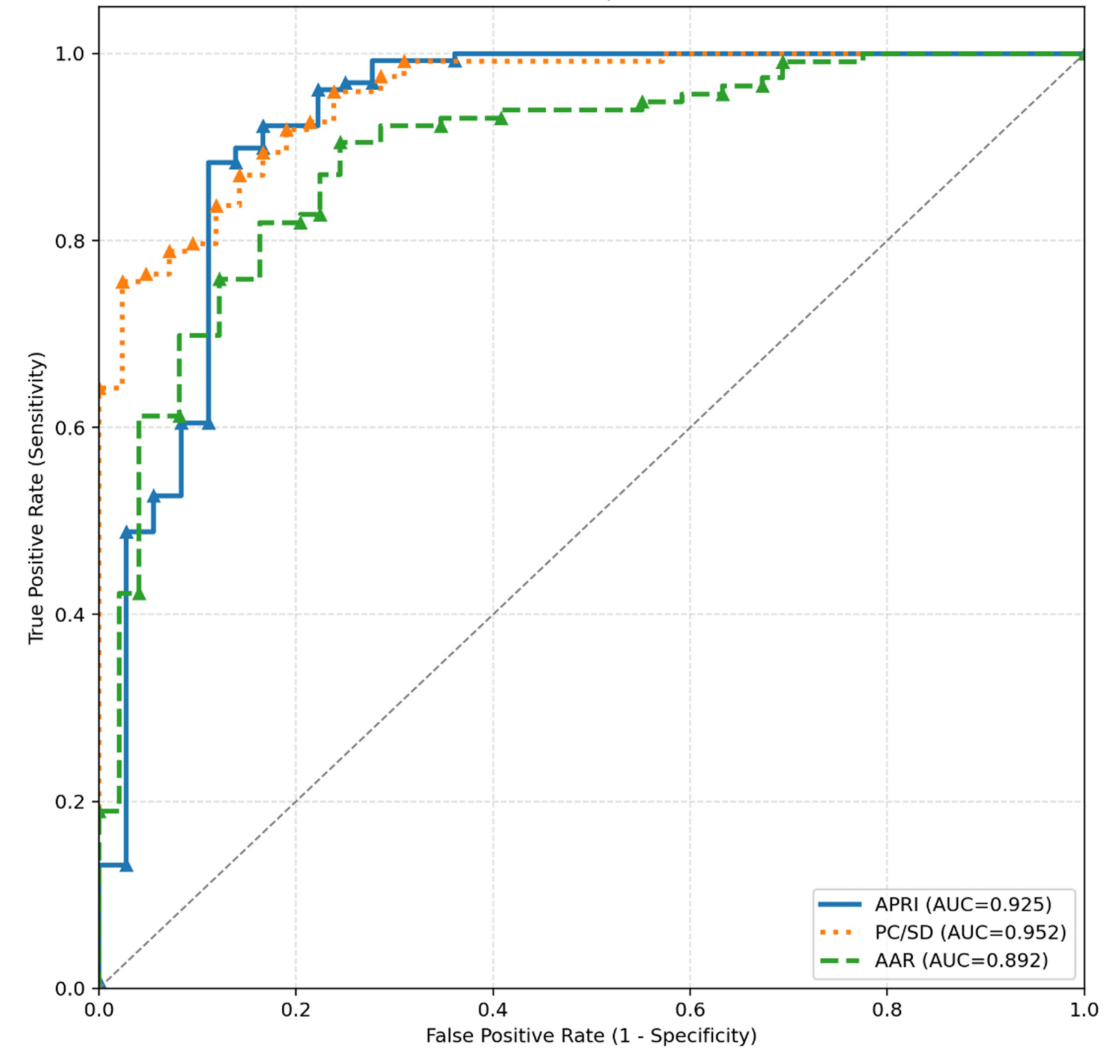
ROC curve of non-invasive markers in the diagnosis of large oesophageal varices Abbreviations: ROC, receiver operating characteristic; APRI, AST-to-platelet ratio index; PC/SD, platelet count/spleen diameter ratio; AAR index, aspartate aminotransferase/alanine aminotransferase ratio.

Among the evaluated non-invasive parameters for predicting large EV, APRI demonstrated excellent discrimination, with an AUC of 0.93 (Table 7). At a threshold of >2.11, APRI had high sensitivity (89%) and good specificity (78.3%), with a high positive predictive value (PPV) of 93.6%, a negative predictive value (NPV) of 63.8%, and diagnostic accuracy (DA) of 83.6%. The PC/SD ratio at a cut-off of ≤ 855 also showed excellent discrimination (AUC, 0.93), with high specificity (91.6%) and PPV (96.6%). An AAR cut-off of >1.2 and platelet count ≤ 41,000 also performed well, with AUCs of 0.92 and 0.92, respectively (Figure 1). Conventional severity scores, including MELD (12.5) and CTP (>11), showed moderate DA. A PV diameter cut-off > 14.5 provided fair discrimination (AUC, 0.79). In contrast, cut-offs for total bilirubin (>2.3mg/dl), serum albumin (≥ 3.1 g/dL), and INR (>2.12) yielded lower AUC values and limited predictive utility. Overall, the selected cut-off values optimized sensitivity and specificity for clinical applicability, and APRI emerged as a reliable non-invasive marker for identifying patients at high risk of large EV. The prevalence of large EV in this cohort was 54.5% (85/156), and this prevalence was used to calculate PPV/NPV for each marker.

**Table 7:** Diagnostic accuracy of non-invasive predictors for large oesophageal varices (Paquet grade III–IV) (n=156) Sample, n=156 (large EV=85; no large EV=71). Large EV is defined as Paquet grade III–IV. Cut-offs are determined from ROC analysis (Youden J). AUC 95% CIs computed using the Hanley–McNeil approximation. Sensitivity, specificity, PPV, and NPV 95% CIs were computed using Wilson intervals. Abbreviations: AAR, aspartate aminotransferase/alanine aminotransferase ratio; APRI, aspartate aminotransferase-to-platelet ratio index; AUC, area under the curve; CTP, Child–Turcotte–Pugh score; DA, diagnostic accuracy; EV, oesophageal varices; INR, international normalised ratio; LC, liver cirrhosis; MELD, Model for End-Stage Liver Disease score; NPV, negative predictive value; PC/SD, platelet count-to-spleen diameter ratio; PPV, positive predictive value; PV, portal vein; Sens, sensitivity; Spec, specificity.

Predictor	Cut-off	AUC (95% CI)	Sensitivity % (95% CI)	Specificity % (95% CI)	PPV % (95% CI)	NPV % (95% CI)	Diagnostic accuracy, %	Youden J
APRI	> 2.11	0.93 (0.89–0.97)	89.4 (81.1–94.3)	78.9 (68.0–86.8)	83.5 (74.6–89.7)	86.2 (75.7–92.5)	84.6	0.683
PC/SD ratio	≤ 855	0.93 (0.89–0.97)	81.2 (71.6–88.1)	91.5 (82.8–96.1)	92.0 (83.6–96.3)	80.2 (70.3–87.5)	85.9	0.727
PV diameter (mm)	> 14.5	0.79 (0.72–0.86)	82.4 (72.9–89.0)	76.1 (65.0–84.5)	80.5 (70.9–87.4)	78.3 (67.2–86.4)	79.5	0.584
AAR	> 1.2	0.92 (0.88–0.96)	87.1 (78.3–92.6)	80.3 (69.6–87.9)	84.1 (75.0–90.3)	83.8 (73.3–90.7)	84.0	0.673
CTP score	> 11	0.83 (0.77–0.89)	85.9 (76.9–91.7)	52.1 (40.7–63.3)	68.2 (58.9–76.3)	75.5 (61.9–85.4)	70.5	0.380
MELD score	> 12.5	0.72 (0.64–0.80)	80.0 (70.3–87.1)	47.9 (36.7–59.3)	64.8 (55.3–73.2)	66.7 (53.0–78.0)	65.4	0.279
Platelet count (×10^3^/µL)	≤ 41,000	0.91 (0.86–0.96)	89.4 (81.1–94.3)	93.0 (84.6–97.0)	93.8 (86.4–97.3)	88.0 (78.7–93.6)	91.0	0.824
Serum albumin (g/dL)	≤ 3.1	0.33 (0.24–0.42)	83.5 (74.2–89.9)	11.3 (5.8–20.7)	53.0 (44.6–61.2)	36.4 (19.7–57.0)	50.6	-0.052
Total bilirubin (mg/dL)	> 2.3	0.53 (0.44–0.62)	69.4 (59.0–78.2)	43.7 (32.7–55.2)	59.6 (49.7–68.7)	54.4 (41.6–66.6)	57.7	0.131
INR	> 2.12	0.65 (0.56–0.74)	78.8 (69.0–86.2)	46.5 (35.4–57.9)	63.8 (54.3–72.4)	64.7 (51.0–76.4)	64.1	0.253

## Discussion

Oesophageal variceal bleeding is among the most serious complications of cirrhosis and is associated with substantial mortality. Approximately 60% to 80% of patients with cirrhosis have EV, and 25% to 35% are at risk of bleeding [29]. This study provides a detailed analysis of demographic, clinical, and laboratory variables in patients with cirrhosis and evaluates the performance of non-invasive predictors for identifying EV.

The study cohort showed a clear male predominance (77.6%), which is consistent with prior reports. However, the sex predominance was not significant across the EV groups. In studies by Duah et al. [30], Gebregziabiher et al. [31], and Sarangapani et al. [11], men accounted for 77.8%, 79.4%, and 74% of participants, respectively. The observed predominance likely reflects differences in exposure to major risk factors for CLD, including alcohol use and viral hepatitis. The mean age of the study population was 45.3 ± 10.4 years, which aligns with findings reported by Duah et al. (45 ± 12.3 years) and Gebregziabiher et al. (41.8 ± 12.4 years) [30,31]. Alcohol use was the most common aetiology in the present study (35.9%), followed by hepatitis B (16.7%), supporting the continued burden of alcohol-related liver disease and hepatitis B virus (HBV) infection in this population. Prior studies from India similarly identified alcohol as the leading aetiology, accounting for 42.4% and 52.6% of cases, whereas chronic HBV infection accounted for 15.3% and 13.1% of cases [8,32]. In contrast, Gebregziabiher et al. reported that HBV infection and schistosomiasis each accounted for 26% of cases [31], and Duah et al. observed alcohol use in 32.9% of cases and chronic HBV infection in 44.3% [30]. Together, these differences suggest that etiologic profiles vary meaningfully by region and clinical setting.

In the present study, grade III varices were the most common (49.36%), followed by grade II (22.44%), and grade IV EV was present in 5.1% of patients. Kumar et al. similarly reported a predominance of grade III varices (44%), although the proportion of grade IV varices was higher (12%) [33]. Naik et al. also reported a higher proportion of grade IV varices (14.02%) compared with the present study [34]. In contrast, Kothari et al. reported a substantially higher prevalence of grade II varices (65.84%), with lower proportions in other grades [35]. These differences may reflect variation in referral patterns, disease stage at presentation, and underlying aetiology across study populations.

Several non-invasive variables were significantly associated with the presence and severity of EV in this study, including serum albumin, total bilirubin, platelet count, INR, aspartate transaminase-to-alanine transaminase ratio (AAR), aspartate transaminase-to-platelet ratio index (APRI), PC/SD ratio, and modified CTP class (P<0.001). However, ROC analyses showed that the PC/SD ratio, AAR, APRI, and platelet count had the strongest discriminatory performance, as reflected by the AUC.

In our study, a PC/SD ratio cut-off of ≤ 855 predicted large EV with a sensitivity of 81.4% and a specificity of 92.2% (AUC = 0.93), regardless of cirrhosis aetiology. Giannini et al. proposed a PC/SD ratio cut-off of ≤ 909 and reported a 100% NPV, supporting its utility as a non-invasive marker for EV [23]. Sarangapani et al. reported that a PC/SD ratio of 909 yielded a PPV of 83.5% and an NPV of 90.5% for EV diagnosis [11]. Cherian et al. reported that a PC/SD ratio of ≤ 666 was significantly associated with EV among patients with alcohol-related cirrhosis [8]. Sen et al. found that a PC/SD ratio of ≤ 650 had an AUC of 0.81 in hepatitis C virus-related cirrhosis but did not perform well in alcohol-related cirrhosis [36]. A low PC/SD ratio likely reflects the combined effects of thrombocytopenia and splenomegaly, both of which are associated with advanced portal hypertension.

Prior studies have reported mixed results for AAR. Iwata et al. observed an association between AAR and EV severity [17], whereas Zhang et al. reported no significant difference in AAR between patients with and without EV and found limited predictive performance (AUC 0.648) [18]. Cifci et al. also reported limited predictive value for AAR in severe EV [37]. In the present study, AAR showed good DA (AUC 0.92) at a cut-off > 1.2, suggesting that higher AAR values are associated with portal hypertension and large EV. However, the modest NPV limits its role as a stand-alone marker to rule out large EV.

For APRI, Zhang et al. reported an AUC of 0.729 and an NPV of 96% for assessing EV severity, with APRI > 1.4 proposed as a useful indicator for early intervention in severe EV [18]. In contrast, our study showed higher discriminative ability, with an AUC of 0.92 and an optimal cut-off of 2.1, yielding PPV and NPV of 92.9% and 72.5%, respectively. These differences may reflect variation in patient characteristics, etiologic distribution, and disease severity across study populations.

In alcohol-related liver disease, the AST/ALT ratio may be elevated independent of portal hypertension because mitochondrial AST release and relative pyridoxine (vitamin B6) deficiency suppress ALT activity. As a result, AST can be disproportionately increased, and the AST/ALT ratio may be higher even at similar levels of fibrosis or portal hypertension. In addition, published evidence suggests that APRI performance is aetiology dependent, with the most consistent validation in hepatitis C cohorts and more variable performance in hepatitis B and alcohol-related disease [38]. Because our subgroup sample sizes were insufficient to derive stable, aetiology-specific cut-offs, we do not claim that the AAR or APRI thresholds observed in this derivation cohort are generalizable across aetiologies.

Thrombocytopenia in LC is commonly attributed to splenic sequestration and destruction, along with reduced hepatic thrombopoietin production. Chalasani et al. identified a platelet count <88,000 as an independent risk factor for large varices [16]. Mahassadi et al. reported that a platelet count cut-off of < 106500 cells/mm³ yielded a DA of 63.7%, a specificity of 80.7%, and a PPV of 84.6% for predicting large EV in Black African patients with cirrhosis [39]. Sarwar et al. also reported platelet count <88000 as an independent risk factor for large varices [40]. Sarangapani et al. reported that a platelet count <150,000/mm³ predicted large varices with a PPV of 63.8% and an NPV of 70.5% [11]. In our study, a platelet count cut-off of 41,000/mm^3^ showed good predictive performance (AUC 0.91) with high PPV, supporting its utility as a rule-in marker for large EV.

The conventional CTP score showed moderate DA in this study (AUC 0.83 at a cut-off of 11). This finding suggests that although the CTP score captures overall liver dysfunction, it performed less well for predicting large EV than APRI, PC/SD ratio, and AAR. Cherian et al. reported that CTP class was the most sensitive marker for detecting large EV (sensitivity 95%, specificity 26%), and CTP class C had the lowest miss rate for large varices (9.5%) [8]. Mujahid et al. reported a significant association (p<0.05) between CTP score severity and the presence of EV [5]. In contrast, Mahassadi et al. found no correlation between CTP class and EV in Ivorian patients with cirrhosis [39], and Sarangapani et al. and Bhattarai et al. similarly reported no association between EV grade and CTP class [11,21]. These inconsistent findings suggest that the relationship between CTP class and EV severity may vary by population and clinical context.

In the present study, the MELD score was significantly associated with EV (p = 0.003) but showed only modest discrimination (AUC 0.669). Kothari et al. reported no association between EV grade and MELD score (p=0.82) [35]. Glisic et al. reported a sensitivity of 70.1% and specificity of 69% for a MELD cut-off of 18 (AUC, 0.637; 95% CI, 0.58-0.69) for assessing variceal bleeding [15]. Taken together, these findings suggest that the MELD score may be associated with EV-related outcomes but has limited utility as a stand-alone predictor of large EV.

In our study, a PV diameter cut-off of >14.5 mm provided balanced sensitivity (82.7%) and specificity (76.3%), with a PPV of 86.8% and an OR of 10.5, supporting PV diameter as a clinically useful ultrasonographic marker for large EV. Uppalapati et al. reported that a PV diameter >13 mm had 100% sensitivity, 90% specificity, and a PPV of 95.2% for predicting EV, and that a larger PV diameter was associated with higher EV grades [20]. Sarangapani et al. similarly reported that PV diameter > 13 mm was significantly associated with large EV (p < 0.001), with sensitivity 92.7%, specificity 90%, PPV 78%, and NPV 78.6% [11]. Bhattarai et al. also reported a correlation between PV diameter and EV grade [21]. These findings support the inclusion of PV diameter as an adjunct marker, particularly when combined with laboratory indices.

In contrast, serum albumin, total bilirubin, and INR showed lower AUC values and limited predictive utility in our analysis, suggesting that isolated markers of hepatic synthetic function are suboptimal predictors of large EV when used alone. Similar findings were reported by Sarangapani et al., Kothari et al., and Cherian et al. [8,11,35]. Taken together, these results support the use of simple non-invasive indices, particularly APRI, PC/SD ratio, and AAR, as adjunct tools for EV risk stratification in resource-limited settings. These findings may support a pragmatic strategy for prioritizing endoscopy in patients at higher risk of large oesophageal varices.

For risk stratification in newly diagnosed cirrhosis, APRI > 2.11, platelet count ≤ 41,000/cu mm, and PC/SD ratio ≤ 855 identified patients at a higher likelihood of large EV in this cohort. When one or more parameters were present, these findings may support earlier endoscopic evaluation, where feasible. Ultrasonographic measures (including PV diameter) may provide additional supportive information when laboratory indices are equivocal.

We defined a lower-risk profile in this cohort as APRI ≤ 2.11, platelet count > 41,000/cu mm, PC/SD ratio > 855, PV diameter < 14.5 mm, and a lower CTP score. Patients meeting these composite criteria had a lower observed likelihood of large EV; however, these criteria should be viewed as derivation findings applicable primarily to similar high-risk settings and require external validation before they are used to alter endoscopy timing in broader clinical populations. We therefore emphasize that these markers are not a substitute for endoscopic examination; rather, they may serve as triage aids when endoscopy resources are constrained. Endoscopy remains essential for definitive diagnosis and management.

Limitations

This study did not confirm the diagnosis of LC using histopathologic evaluation from liver biopsy, which is considered the gold standard. Operator dependence and interobserver variation may have affected ultrasonographic measurements, including spleen size and PV diameter. These findings require validation in larger cohorts.

In addition, this was a single-centre study conducted in an industrial hospital, and the patient population and referral patterns may differ from those of community hospitals or tertiary care centres. As a result, the observed prevalence and severity distribution of EV and the performance of specific cut-offs may not be generalizable to other regions or practice settings. Although the study was prospective, the observational design limits causal inference and does not allow assessment of whether applying these predictors in routine care would reduce bleeding events or improve clinical outcomes. We used the Paquet grading system, which focuses primarily on variceal size; however, we did not systematically record or analyze red colour signs such as red wale marks, which are important for defining ‘clinically significant’ or ‘high‑risk’ varices according to current consensus guidelines (e.g., the Baveno recommendations).

The prevalence of EV in our cohort was high, with relatively few patients without varices. This likely reflects the referral-based nature of enrolment and a greater representation of decompensated cirrhosis. Because PPV and NPV depend on disease prevalence, PPV in this cohort may be inflated, and NPV may be reduced compared with cohorts that include more compensated cirrhosis or population-based screening samples. Accordingly, the proposed cut-offs should be interpreted as tools for risk stratification in settings with a similar case mix and should be externally validated before they are used to defer endoscopy in broader clinical populations.

Several predictors included in the models are biologically and statistically related (e.g., platelet count, splenomegaly, and PC/SD ratio), which may introduce collinearity and could affect coefficient estimates in multivariable analyses. The study also relied on routinely collected clinical data and did not standardize factors that can influence platelet count, liver biochemistries, and coagulation parameters, including intercurrent infection, recent alcohol intake, medication effects, or nutritional status. Finally, although this work identified cut-offs with favourable diagnostic performance, the study did not include external validation or a predefined calibration assessment, and prospective implementation studies are needed to confirm the performance, feasibility, and safety of endoscopy deferral strategies based on these non-invasive criteria.

## Conclusions

In this study evaluating non-invasive predictors of large EV in patients with LC, several readily available clinical, laboratory, and ultrasonographic variables showed useful diagnostic performance. APRI and the PC/SD ratio demonstrated the highest discriminatory ability, with excellent AUC values, sensitivity, specificity, and overall DA, supporting their potential utility as triage tools for large EV. In this referral-based cohort, these thresholds may assist with prioritizing endoscopy in similar high-risk settings; however, external validation is needed before applying these cut-offs to populations with more compensated cirrhosis. PV diameter also showed fair discrimination and may serve as a practical ultrasonographic marker of portal hypertension. The AAR, modified CTP score, and MELD score showed moderate accuracy, whereas isolated biochemical measures such as serum albumin, total bilirubin, and INR had limited predictive value when used alone. Though these findings suggest that non-invasive indices and imaging measurements can help stratify patients with cirrhosis who are at risk of large EV, given the advanced case‑mix and high disease prevalence in this cohort and the shared biological components across several indices, these operating points should be interpreted as setting‑specific derivation thresholds rather than general screening rules, and endoscopy remains essential for definitive diagnosis and management.
